# Trade-offs between deer herbivory and nitrogen competition alter grassland forb composition

**DOI:** 10.1007/s00442-023-05485-9

**Published:** 2023-12-13

**Authors:** George N. Furey, David Tilman

**Affiliations:** 1 Faculty of Environmental Sciences and Natural Resource Management, Norwegian University of Life Sciences, Universitetstunet 3, 1433 Ås, Norway; 2 Department of Ecology, Evolution and Behavior, College of Biological Sciences, University of Minnesota, St. Paul, MN USA; 3 Bren School of Environmental Science and Management, University of California, Santa Barbara, CA USA

**Keywords:** *Artemisia ludoviciana*, Competition-defense, Florivory, *Solidago rigida*, White-tailed deer

## Abstract

Two of the major factors that control the composition of herbaceous plant communities are competition for limiting soil resources and herbivory. We present results from a 14-year full factorial experiment in a tallgrass prairie ecosystem that crossed nitrogen (N) addition with fencing to exclude white-tailed deer, *Odocoileus virginianus*, from half the plots. Deer presence was associated with only modest decreases in aboveground plant biomass (14% decrease; −45 ± 19 g m^−2^) with no interaction with N addition. N addition at 5.44 and 9.52 g N m^−2^ year^−1^ led to increases in biomass. There were weak increases in species richness associated with deer presence, but only for no or low added N (1 and 2 g N m^−2^ year^−1^). However, the presence of deer greatly impacted the abundances of some of the dominant perennial forb species, but not the dominant grasses. Deer presence increased the abundance of the forb *Artemisia ludoviciana* by 34 ± 12 SE g m^−2^ (94%) and decreased the forb *Solidago rigida* by 32 ± 13 SE g m^−2^ (79%). We suggest that these changes may have resulted from trade-offs in plant competitive ability for soil N versus resistance to deer herbivory. Field observations suggest deer acted as florivores, mainly consuming the flowers of susceptible forb species. The preferential consumption of flowers of forbs that seem to be superior N competitors appears to create an axis of interspecific niche differentiation. The overpopulation of white-tailed deer in many tallgrass reserves likely structures the abundance of forb species.

**Supplementary Information:**

The online version contains supplementary material available at 10.1007/s00442-023-05485-9.

## Introduction

Herbivory and resource competition can jointly influence the structure of plant communities (Huntly 1991; Chase et al. 2002; Aschehoug et al. 2016) and determine whether interspecific interactions among plants lead to competitive exclusion or coexistence (Holt et al. 1994). Ecologists have long sought to understand the mechanisms underpinning exploitative resource competition (Hutchinson 1959; Tilman 1982, 2011; Chesson 2000; Grace and Tilman 2003). Other theory has explored how herbivory could impact coexistence in the absence of direct competition, but indirectly via apparent competition (Holt and Kotler 1987; Holt and Bonsall 2017). A combination of both exploitative and apparent competition suggests that an inferior competitor can coexist with a competitively dominant species in the presence of an herbivore (Holt et al. 1994). Because plants simultaneously experience both herbivory and exploitative competition in natural food webs, it can be difficult to assess both their interactive and independent impacts on the outcome of plant competition. The joint effects of exploitative competition and herbivory on a plant community, though, may be revealed by factorial experimental designs that cross the addition of limiting nutrients with the presence/absence of an herbivore. Here we report results of a long-term experiment in a tallgrass prairie ecosystem in which various addition rates of the limiting nutrient, nitrogen (N), were fully crossed with a fencing treatment that excluded the only large mammalian herbivore remaining in the ecosystem, white-tailed deer, *Odocoileus virginianus*.

Herbivory has been shown to impact plant diversity, but the results often depend on both the type of herbivore and its feeding preferences. Herbivory may increase plant diversity when an herbivore consumes a competitively dominant species (Hillebrand et al. 2007; Koerner et al. 2018). The consumption of biomass increases light availability and can thus reduce light competition. This is a possible way that herbivory could promote plant diversity (Borer et al. 2014; Eskelinen et al. 2022). From the perspective of resource competition theory, the decrease in abundance of a dominant competitor can act as a coexistence mechanism if plants have interspecific trade-offs in competitive ability versus resistance to herbivory (Levin et al. 1977; Holt et al. 1994; Viola et al. 2010). For instance, if a trade-off were to exist between competitive ability for soil nitrate and resistance to herbivory, species that are most abundant in plots with no added N, and thus are likely stronger competitors by drawing soil nitrate to a lower level (R*), should be more helped by fencing as they would be poorly defended because of the trade-off (Holt et al. 1994). Conversely those species that increase with added N (which should be poorer N competitors) should increase in the absence of fencing as they are better defended and have a lower R* in the presence of herbivory (denoted R**) (Holt et al. 1994). It is not clear however, if and under what conditions, these theoretical predictions hold empirically since one often-reported outcome has been a trade-off between growth and defense (Coley et al. 1985; Fine et al. 2004, 2006; Viola et al. 2010; Lind et al. 2013).

Herbivory is often selective with an herbivore preferentially consuming different plant species depending on their traits. A simple heuristic is that grazers often focus on grasses whereas browsers often focus on herbaceous non-grasses, hereafter forbs (Gordon and Herbert 2019). Numerous studies have shown that grazers can have large impacts on species within family *Poaceae* (McNaughton 1985; Augustine and McNaughton 1998; Knapp et al. 1999; Towne et al. 2005). In contrast, browsing, such as by white-tailed deer, often appears to remove forbs, which may benefit their grass competitors (Anderson et al. 2005; Wiegmann and Waller 2006; Rooney 2009). Such browsing preferences may have cascading ecosystem consequences when deer selectively target species or plant tissues with higher N concentrations, such as N-fixing species, and thus reduce total system N availability and influence species' competitive interactions for N (Ritchie et al. 1998).

Because different mammalian herbivores may consume different types of plant tissues, (Gordon and Herbert 2019), each form of herbivory might uniquely impact plant species abundances. Florivory, which represents a direct consumption of flowers that may limit reproductive success (Anderson et al. 2001; McCall and Irwin 2006; Geddes and Mopper 2006), has received limited treatment in ecological theory and experiments. However, a serendipitous event during an herbivory experiment alerted us to its potential importance. A white-tailed deer that broke through fencing was observed to have preferentially consumed flowers of certain forb species. Further quantification of florivory that we report provides insight into how and why deer impact grassland plant competition and species abundances. Although white-tailed deer florivory on herbaceous species has been reported (Augustine and Frelich 1998; Anderson et al. 2001, 2007; Geddes and Mopper 2006; Flaherty et al. 2018; Palagi and Ashley 2019), there is less information on how florivory may interact with resource competition in structuring herbaceous plant communities. Here we explore the possibility that florivory may be an important component of interspecific trade-offs related to competition, growth and defense against herbivores (Coley et al. 1985; Holt et al. 1994; Viola et al. 2010; Lind et al. 2013).

Our objective was to test how excluding or not excluding deer with fences from plots in a long-term nutrient addition experiment impacted aboveground plant biomass, plant biodiversity and species composition. A full factorial design allowed us to measure the joint and independent impacts of herbivory and N addition on a plant community. All plots had first been fenced to exclude white-tailed deer for 22 years (1982–2004) and then 27 plots were changed to be unfenced for 14 years (2005–2019). The resultant data test how both the presence/absence of white-tailed deer and different rates of N addition influenced total aboveground plant biomass, plant biodiversity and plant species' abundances. We also used these data to determine if the dominant plant species exhibited trade-offs like those assumed for a competition–defense (Holt et al. 1994; Viola et al. 2010; Petry et al. 2018) or growth–defense trade-off (Coley et al. 1985; Fine et al. 2004, 2006; Lind et al. 2013). Lastly, we mention some opportunistic observations of deer browsing to gain an insight into this specific form of herbivory.

Our research questions were therefore:

1. How do (a) total aboveground plant biomass and (b) plant species richness depend on N addition, on the presence/absence of a deer exclosure, and on their interaction?
  Our expectation for the main effect of each treatment was that the removal of fences would lower total aboveground biomass and increase plant species richness. We expected that the addition of N combined with the removal of fences would increase the production of plant biomass through a compensation mechanism. We expected that the removal of fences would counteract the decrease in species richness associated with increased added N.
2. Is the abundance response of individual plant species to N addition and fencing removal consistent or inconsistent with a trade-off between N-dependent competitive ability and resistance to herbivory?
  Our expectation was that there would be a trade-off among plant species such that those species that decreased with added N (presumed to be better N competitors) would benefit from fencing, whereas those species that increased with added N would be more abundant outside the fence (where deer could consume their competitors).
3. Which species had detectable incidences of florivory, and on a species-by-species basis does a greater proportion of florivory correspond with a lower abundance of a species in the presence of deer (unfenced plots)?
  Our expectation was that fencing promotes the abundance of forbs susceptible to deer browsing and that these species would also have detectable incidences of deer browsing their flowers outside the fence.

## Methods

### Site description

The experiment was conducted at the Cedar Creek Ecosystem Science Reserve, Minnesota, USA. The site has a sandy soil with a particle size distribution of ~90% sand (Udipsamments) that is nutrient poor with low organic carbon (Grigal 1974). The field is located at 45.397334°, −93.191648° and referred to as "Field C" within the experiment "e001: Long-Term Nitrogen Deposition: Population, Community, and Ecosystem Consequences". The field was abandoned from maize row crop agriculture in 1934.

### Experimental design

The experiment consists of 54 plots sized 4 by 4 m with 1 m buffers between them arranged in a 9 × 6 rectangular grid. In 1982, each of 54 plots in an area were fenced to exclude white-tailed deer and were randomly assigned to receive nutrient treatment of either no added nutrients of any kind, or of one of eight levels of added N (0.0, 1.02, 2.04, 3.4, 5.44, 9.52, 17.0, 27.2 g N m^−2^ year^−1^) plus nutrients P, K, Ca, Mg, S and trace metals (Tilman 1987). Two sets of plots received no added N: Treatment I which received no nutrients of any kind and Treatment A which received no N, but all other nutrients. Treatment A serves as the control to test solely for the effect of N addition, whereas Treatment I serves as the control for nutrient addition of any kind.

The fertilizer addition was conducted as follows. Each plot received N as ammonium nitrate with an N content of 34%. In early May and again in late June of 1982 and each subsequent year, one-half of the annual amounts of all nutrients for each plot were mixed together and then manually broadcast on each plot. Each treatment received the following amounts of ammonium nitrate twice each year (0.0 = 0 g m^−2^; 1.02 = 1.5 g m^−2^; 2.04 = 3 g m^−2^; 3.4 = 5 g m^−2^; 5.44 = 8 g m^−2^; 9.52 = 14 g m^−2^; 17.0 = 25 g m^−2^; 27.2 = 40 g m^−2^). Additionally, all treatments except for the true control (Treatment I) received twice annually 10 g m^−2^ year^−1^ P_2_O_5_; 10 g m^−2^ year^−1^ K_2_O; 15.0 g m^−2^ year^−1^ MgSO_4_; 20 g m^−2^ year^−1^ CaCO_3_; 18.85 µg m^−2^ year^−1^ ZnSO_4_; 9 µg m^−2^ year^−1^ CuSO_4_; 7.65 µg m^−2^ year^−1^ 161 µg m^−2^ year^−1^ MnCl_2_; CoCO_2_; 7.55 µg m^−2^ year^−1^ NaMoO_4_. Sheet metal was installed in between all plots to a depth of 30 cm to prevent root foraging for nutrients between plots and fertilizer contamination.

A deer herbivory by nutrient addition experiment was imposed in 2005 on the existing nutrient addition experiment that had been established in 1982. All plots had been fenced to exclude deer from 1982 to 2004. The experimental design is a full factorial with nine nutrient treatments, two levels of fencing, and three replicates of each of the 18 treatment combinations (though a subset of nutrient treatments were considered, as described below). The experiment was initiated in fall of 2004 by removing the fence that had surrounded the full experiment. After fencing removal, three of the six replicates for each of the nine nutrient addition treatments were randomly chosen to be re-fenced. The other three replicates of each nutrient treatment were unfenced. Randomization was repeated a few times until the resulting spatial arrangement of fencing assured that deer could freely enter all unfenced plots. The mesh size of the fence was large enough to not exclude small mammals, but whenever a plains pocket gopher, *Geomys bursarius*, entered a plot it was trapped and removed. The removal of fencing coincided with the initiation of an annual burning of all plots of this experiment in the fall of 2004. We do not know if the field experienced one or more regional wildfires following abandonment from agriculture in 1934. It was not burned from 1982 to 2004 and has been annually burned in early spring since 2005, which is the same year that this nutrient addition treatment crossed with fencing treatments began. The layout of the experiment is presented in Fig. S1.

### Sampling

Vegetation in each plot was annually sampled by clipping, then sorting to species, drying and weighing the aboveground plant biomass in a 10 cm × 3 m strip. Clip strips were located so as never to be adjacent to any area that had been clipped within the last 5 years, and to never re-clip any area that had been clipped during the past 15 years. Plant abundances in all plots were sampled in August of 2005, the first growing season for this herbivory × nutrient addition experiment, and in 10 of the subsequent 14 years from 2006 to 2019 (not clipped in 2012, 2013, 2016, and 2017). Vegetation sampling used the same protocol as had begun in 1982. Due to the annual burning, woody species in this field are rare in the annual surveys. An adventitious woody stem is occasionally clipped in a few plots, but too rarely to provide information on the potential impacts of deer and therefore woody species were not included in our analyses.

We conducted a separate estimate of deer herbivory across all plots in August of 2016. This sampling was initiated following an observation that a white-tailed deer, identified through the presence of its tracks and feces, broke through fences enclosing plots 25, 26, 31, 32 and 37 sometime between July 3rd and 4th, 2016. Following an informal visual survey for signs of vegetation consumption, the fence was repaired. A separate survey was conducted across all experimental plots in August during peak biomass in this ecosystem when the plots are normally clipped. To formally estimate the extent that deer florivory (flower consumption) could be occurring across all plots in the experiment, we used a 4 m × 0.5 m quadrat in each plot randomly assigned to the east or west side of each plot. To allow comparison across different inflorescence structures, each stem with a visible inflorescence in any stage of anthesis was counted. We recorded deer florivory as the removal of a whole inflorescence below the peduncle that could be visually compared to an intact version within the fencing treatment where possible. In each transect, the total number of stems per each species was estimated and we recorded how many of these stems had a removed inflorescence.

### Data analyses

Analyses used R version 4.1.1, with experimental variables being the crossed fencing and nutrient addition treatments. We refer to the fencing treatment as "UnFenced" and "Fenced". We report analyses on a subset of experimental treatments. We did not consider the two highest levels of N addition (as explained by Clark and Tilman 2008) because these rates are biologically unrealistic (270% and 490% above in situ N mineralization rates; Pastor et al. 1987), causing plant die-offs, invasions by exotic annuals and extreme biomass oscillations. We additionally used only those treatments that received all potentially limiting nutrients along the gradient of N addition excluding the true controls (Treatment I). We did this because theoretical models of resource competition and herbivory often assume there is only one limiting nutrient (Tilman 1982; Holt et al. 1994), which is achieved experimentally by adding all potentially limiting nutrients except N (a P, K, Ca, Mg+ fertilizer mix) to all plots. We sought to test solely for the effect of N addition. We identify these treatments by their annual N addition rates of 0.0, 1.02, 2.04, 3.4, 5.44, 9.52 g m^−2^ year^−1^ of N. The maximum N treatment in this subset then approximates a doubling of the background soil net N mineralization rate. We compare plots that did not receive N (Treatment A) to the various N addition treatments, all in the set of plots that received P, K, Ca and Mg+ fertilizer. The sample size for our analyses is 36 plots (36 plots = 6 nutrient treatments × 2 fence treatments × 3 replicates).

#### Question 1a:

Does total aboveground plant biomass depend on N addition, deer exclosure or their interaction?

Aboveground plant biomass was calculated as the sum of live aboveground herbaceous biomass (g m^−2^). The dependence of aboveground biomass on a fully crossed interaction with the natural log of year as a linear continuous variable (2005–2019), N addition as a categorical variable (6 levels), and fencing as a categorical variable (2 levels) was determined using a linear mixed effects model (*nlme*) (Pinheiro and Bates 2000)—n.b. only years post-fencing removal were analyzed. Plot was included as a random intercept. A separate variance term was included for both the effect of fencing and N treatments to account for unequal variance, to address heteroscedasticity and to improve the model fit to the data (*nlme::varIdent*). We additionally tested a log-transformed *y*-variable, but it reduced the fit to the data. A compound symmetry temporal autocorrelation structure was included. We tested the significance of the two-way and three-way interactions using a nested likelihood ratio test. We present the full model with all interactions in Table 1. Pairwise tests across the fencing treatment were also run on the unconditional main effect without insignificant interactions, because higher-level interactions were not supported based on likelihood ratio tests.

**Table 1:** Summary ANOVA table testing the dependance of total live aboveground biomass and the number of herbaceous species on the fencing treatment as a categorical variable, the nitrogen addition treatment as a categorical variable and year as a continuous variable or the natural log of year for the model for biomass for 11 years of data (2005–2019, not including 2012, 2013, 2016, 2017). *n* = 36

Term	numDF	denDF	Aboveground biomass		Species richness	
			*F* value	*P* value	*F* value	*P* value
Fencing treatment	1	24	3.5	0.073	11.95	0.002
Nitrogen treatment	5	24	0.53	0.754	2.75	0.042
Year	1	348	19.89	< 0.001	2.53	0.112
Fencing treatment × Nitrogen treatment	5	24	1.46	0.240	3.91	0.010
Fencing treatment × year	1	348	3.51	0.062	12.0	0.001
Nitrogen treatment × year	5	348	0.53	0.757	2.78	0.018
Fencing treatment × nitrogen treatment × year	5	348	1.46	0.203	3.91	0.002

#### Question 1b

Does plant species richness depend on N addition, deer exclosure or their interaction?

Species richness was calculated as the number of observed vascular herbaceous species in each plot and excluded occasional stems of woody shrubs. The same model selection procedure was used as for the model for aboveground biomass and the same specification except with a linear term for year that improved the model fit. Given a significant three-way interaction among year, N addition and fencing, the difference among slopes through time across the fencing treatment at each level of N was tested using package *emmeans* with *P* values corrected using a Tukey correction (Lenth 2020). Each slope was tested if it differed from zero and corrected using a Bonferroni correction (Lenth 2020).

#### Question 2

Is the abundance response of individual plant species to N addition and fencing removal consistent or inconsistent with a trade-off between N-dependent competitive ability and resistance to herbivory?

The dependance of each individual plant species' abundance (aboveground biomass g m^−2^) on the natural log of year as a linear continuous variable, the effect of N as a linear continuous variable and the fencing exclosure as a categorical variable (two levels) were tested using a linear mixed effects model. We used N as a continuous linear variable to report effect size of biomass per g of added N and it often gave a more parsimonious fit than the natural log of added N. N was treated as a continuous variable to provide one number as an effect size to use as a proxy for N competitiveness. Plot was included as a random intercept. A separate variance term was included for both experimental variables to account for unequal variance, to address heteroscedasticity and to improve the model fit to the data (*nlme::varIdent*). *P* values were adjusted using the false discovery rate correction (Benjamini and Hochberg 1995). The top ten most abundant grass, legume and forb species were tested representing ~ 88% of the total aboveground biomass. Models for the species less abundant than these frequently failed to converge. We dropped the first 2 years after the fences were removed. Species abundance patterns strongly displayed transient dynamics, such as when: "[a] system can undergo complex dynamics during the transition from its original state to the new experimentally imposed state (Tilman 1989)." This exclusion was necessary at the level of individual species as there was carryover effects from pre-treatment fencing conditions that took several years to realize post-fencing removal. Additionally, to simplify the statistical models for each species and the effect size we report, we did not include year × fencing interactions for each species as we did for both total biomass and species richness. Where we used the mean response to fencing removal across the years included, we seek to report the long-term outcome of the fencing removal perturbation. Years included are therefore 2007–2019 (not clipped in 2012, 2013, 2016, and 2017).

The effect size for N and the effect size for fencing on each individual species’ biomass were tested for correlation using major axis regression (Lind et al. 2013). In this manuscript, we denote species in family *Poaceae* such as grasses (*n* = 4), species in family *Fabaceae* such as legumes (*n* = 1), and the remaining species such as forbs (Families: *Euphorbiaceae* *n* = 1; *Asteraceae* *n* = 4).

#### Question 3

Which species had detectable incidences of florivory, and on a species-by-species basis does a greater proportion of florivory correspond with a lower abundance of a species in the presence of deer (unfenced plots)?

The proportion of inflorescences removed per each species is presented as supplemental data to support the statistical models of each species' abundance. We used the total number of browsed inflorescences divided by the total number of counted inflorescences to calculate a proportion. The hypothesis that the proportion of browsed inflorescences was equal to zero was tested using a two-proportion *Z*-test (*prop.test*) with *P* values adjusted using the false discovery rate.

## Results

**Fig. 1:**
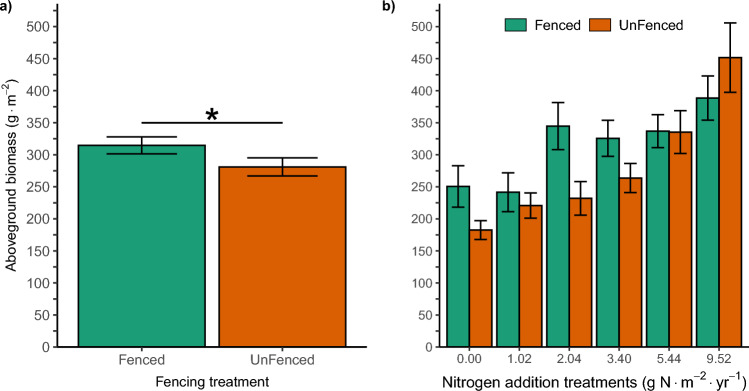
**a** Plant total live aboveground biomass across fencing exclosure treatments. Observed mean total aboveground biomass (g m^−2^) ± 1 SE averaged across N addition treatments 0.0–9.52 g N m^−2^ year^−1^ for each fencing treatment (*n* = 18). **b** Observed mean total aboveground biomass (g m^−2^) ± 1 SE in N addition treatments 0.0–9.52 g N m^−2^ year^−1^ for each fencing treatment (*n* = 3 within each fencing treatment for each level of N). For both panels years included 2005 through 2019 (not clipped in 2012, 2013, 2016, and 2017)

There was no three-way interaction between the natural log of year, fencing and added N in the full statistical model for total live aboveground biomass (Table 1). Biomass increased with added N, but with no interaction with fencing (Fig. 1b). A fencing × N interaction was not retained following model selection (*P* = 0.27). Following model selection, biomass depended on main effects for added N, fencing and the natural log of year (Table S1). Plots outside the fence had less biomass (44.6 ± 19.4 SE, *df* = 29, *P* = 0.0292) on average across the N treatments of 0.0 g N–9.52 g N m^−2^ year^−1^ (Fig. 1a). The main effect for N reveals a gain in biomass relative to the control on average across the fencing treatment of 0.0 g N m^−2^ year^−1^ at 5.44 g N m^−2^ year^−1^ (126 ± 32.8 SE, *P* = 0.0247) and 9.52 g N m^−2^ year^−1^ (216.2 ± 46.7 SE, *P* = 0.0072) (Table S2).

**Fig. 2:**
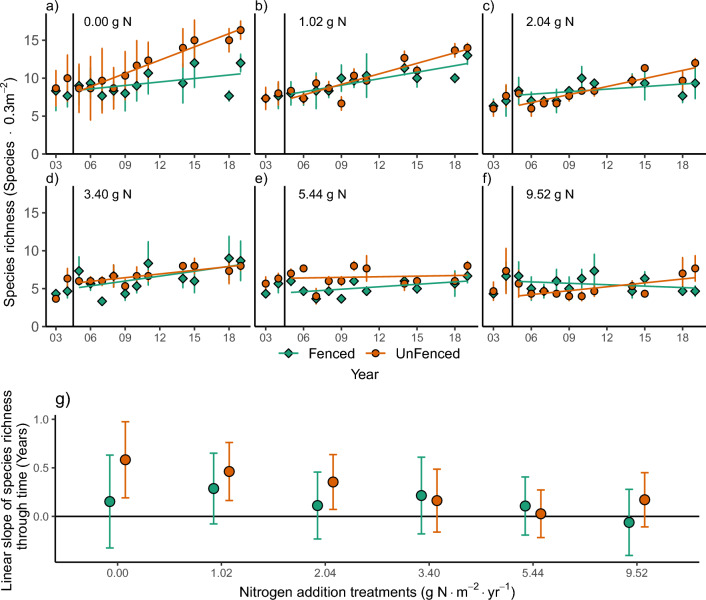
**a–f** Trends in plant species richness (2003–2019), for each nitrogen addition treatment (g m^**−**2^ N year^**−**1^), showing deer excluded plots and unfenced plots. Each point represents the observed mean of the number of species for each nitrogen treatment at each fencing treatment (*n* = 3 within each level of added N). The lines represent fitted values from a linear mixed effects model testing the dependance of the number of species on a three-way interaction between the categorical fencing treatment, the added nitrogen treatment as a categorical variable and year as a linear continuous variable (> 2004). The vertical bar denotes when the fences were removed in the fall of 2004 and deer could enter half the plots freely. Two pre-treatment years are shown in 2003 and 2004 (but not included in the model) followed by the years with fences removed 2005–2019 excluding 2012, 2013, 2016, and 2017. Nitrogen addition treatments: **a** 0.00 g N m^**−**2^ year^**−**1^, **b** 1.02 N g m^**−**2^ year^**−**1^, **c** 2.04 g N m^**−**2^ year^**−**1^, **d** 3.40 g N m^**−**2^ year^**−**1^, **e** 5.44 g N m^**−**2^ year^**−**1^, **f** 9.52 g N m^**−**2^ year^**−**1^. *n.b.* all treatments also received all other limiting nutrients as fertilizer. **g** The slope through time ± a 95% confidence interval for each fencing × nitrogen treatment testing the dependance of species richness on year as a linear continuous variable

The removal of fences increased plant species richness, but only in plots where N was not added or was added at low amounts. Species richness depended on a significant three-way N by fencing by year interaction (*P* = 0.002) (Table 1). The slope through time outside the fence at 0, 1.02 and 2.04 g N m^−2^ year^−1^ were different from zero with slopes of 0.58 (95% C.I. [0.19, 0.98], *P* = 0.0085), 0.46 (95% C.I. [0.16, 0.76], *P* = 0.007) and 0.35 (95% C.I. [0.072, 0.64], *P* = 0.019), respectively, but none of these slopes differed from each other (Fig. 2e). Furthermore, the fencing treatment had no detectable effect on the trend in species richness through time when N was added at a rate of 3.4, 5.44 or 9.52 g N m^−2^ year^−1^ (Fig. 2).

Of the ten most abundant grass, legumes and forb species, the removal of fencing changed the abundances of five forb species, but only of one grass species. Across all N treatments, forbs *Solidago rigida, Symphyotrichum oolentangiense* (formerly *Aster azureus*) and *Euphorbia corollata* decreased outside the fence with an effect size of the fencing treatment from 2007 to 2019 of 32 ± 13 SE g m^−2^, 5.8 ± 2.5 SE g m^−2^ and 6.9 ± 1.9 SE g m^−2^ of biomass, respectively (*P* = 0.045, *P* = 0.045, *P* < 0.001, with effect sizes representing 14%, 2.6% and 3.1% of the mean total live aboveground biomass in plots receiving no added N). In contrast, the biomass of forbs *Artemisia ludoviciana* and *Ambrosia coronopifolia* increased by 34 ± 12 SE g m^−2^ and 7.9 ± 2.6 SE g m^−2^, respectively, outside the fence (*P* = 0.027, *P* = 0.023, with effect sizes representing 15% and 3.5% of the mean total live aboveground biomass in plots receiving no added N) (Table S3). The biomass of legume *Lathyrus venosus* decreased outside the fence, but not significantly, by 6.4 ± 3.3 SE g m^−2^ (*P* = 0.086). The fencing treatment had a biomass of C3 grass *Panicum oligosanthes*, with 3.7 ± 1.6 SE g m^−2^ more biomass outside the fence (*P* = 0.045, with an effect size representing 1.6% of the mean total live aboveground biomass in plots receiving no added N) (Table S3).

As to N addition, the forb *S. rigida* decreased with an effect size of N addition from 2007 to 2019 of 4.7 ± 1.9 SE g m^−2^ per g m^−2^ of annual N addition (*P* = 0.039), whereas *A. ludoviciana* increased by 13.9 ± 2.4 SE g m^−2^ per g N m^−2^ year^−1^ (*P* < 0.001) (Table S3. The grass *Sorghastrum nutans* decreased by 0.44 ± 0.18 g m^−2^ per g N m^−2^ year^−1^ (*P* = 0.039), whereas grasses *Poa pratensis* and *Elymus repens* increased by 7.1 ± 1.5 SE and 11 ± 3.6 SE g m^−2^ per g N m^−2^ year^−1^ (*P* < 0.001; 0.018) (Table S3).

**Fig. 3:**
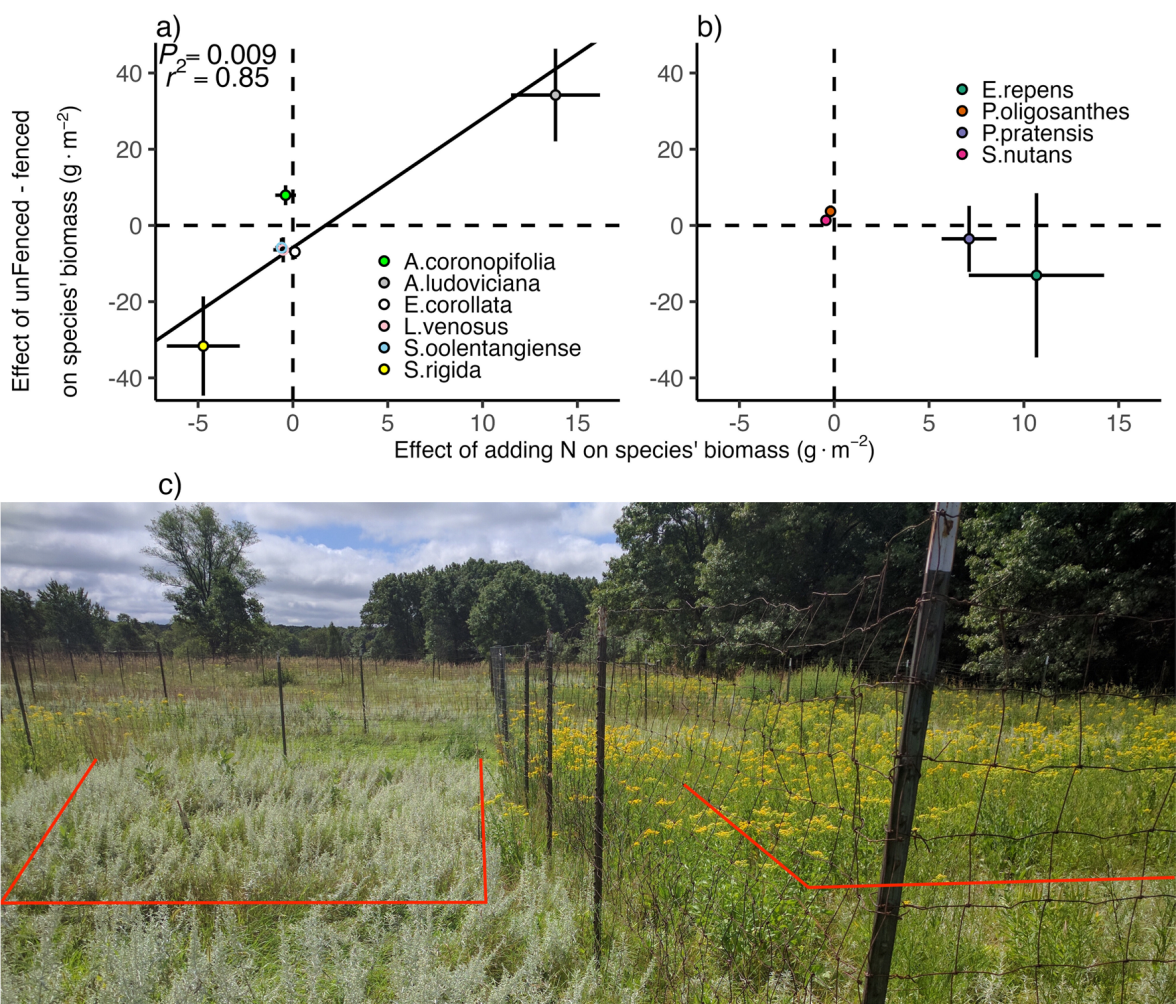
The effect size of a separate linear mixed effects model for each of **a** forb and legumes and **b** grass species testing the dependence of an individual species' biomass on nitrogen (linear variable) and fencing (categorical variable). A positive value on the *y*-axis means that a species is more abundant when in an area that is not fenced, i.e., when deer could be present. The *x*-axis shows the effect of N addition on the abundance of a species, i.e., the slope of a regression for a species' abundance on the rate of N addition. Each point represents the coefficient in each species’ statistical model ± 1 SE from Table S3. The fitted line represents a major axis regression displaying the relationship between both variables for forbs and legume species **c** Picture of treatment plots 9 (9.52 g N m^−2^ year^−1^ unfenced; left) and plot 8 (0 g N m^−2^ year^−1^; right). Note the silver-gray foliage and flowers of *Artemisia ludoviciana* and the yellow flowers of *Solidago rigida*. Photo from 08-Aug-2016 illustrates dominance by *S. rigida* inside the fence in plots with no added N contrasted with dominance by *A. ludoviciana* outside the fence at high added N. Red lines denote the approximate position of the two 4 × 4 m plots positioned using the plot “9” tag and posts visible on the left

We used the effect sizes from regressions of the dependence of each species' biomass on N and fencing to determine if there might be a trade-off between competition for N and benefitting from the removal of fences. Using the effect sizes of all six forbs and legumes, our results show a trade-off (Fig. 3), based on a major axis regression with a positive slope of 3.38 g of biomass gained outside the fence for each gram of added N (*r*^2^ = 0.85, *P* = 0.009) (Fig. 3a). In contrast, only one grass species, *P. oligothanses*, changed in abundance with the fencing treatment (Fig. 3b).

A visual survey of florivory on individuals of these species indicated that several forb species had been browsed, but no grasses had any evidence of deer florivory. A *Z*-test on the percentage of browsed inflorescences indicated that four species had detectable levels of florivory: *S. oolentangiense* (56.2% 45/80)*, E. corollata* (51.7% 30/58),* S. rigida* (30.3% 20/66), and *L. venosus* (47.3% 70/148) (all *P* < 0.001). Although these counts of florivory events came from one season’s field observations, all the species with detectable deer florivory also had less biomass outside the fence: *S. oolentangiense* 5.8 ± 2.5 SE g m^−2^, *E. corollata* 6.9 ± 1.9 SE g m^−2^, *S. rigida* 32 ± 13 SE g m^−2^. *L. venosus* did not significantly change in biomass from the fencing treatment (6.4 ± 3.3 SE g m^−2^ less biomass outside the fence) given the sample size and variance (but see Ritchie and Tilman 1995; Ritchie et al. 1998; Knops et al. 2000).

## Discussion

When compared to fenced plots, unfenced plots, which allowed entry by white-tailed deer, caused declines in total live aboveground biomass and increases in plant species richness in this grassland ecosystem. Unfenced plots had a ~ 14% (45 g m^−2^) decrease in plant biomass, comparable in magnitude to the increase in biomass associated with the addition of ~ 1–2 g N m^−2^ year^−1^ (Table S2). The increase in biomass of the forbs *A. ludoviciana* and *A. coronopifolia* after the removal of fencing appears to have mostly offset the decrease in biomass of *S. rigida, E. corollata* and *S. oolentangiense*.

### Species’ responses to the experimental treatments

The most interesting impacts of the fencing treatment were changes in the abundance of several forb species. *S. rigida* was highly abundant when N was the sole limiting nutrient inside the fence. In contrast, *A. ludoviciana* was highly abundant when N and all other nutrients were highly available outside the fence. The responses of common grassland forbs to the experimental treatment suggests interspecific trade-offs between competitive ability for soil N and susceptibility to deer consumption. If deer are florivores of some grassland forbs, then the flowers so removed may be a small proportion of the total biomass of these plots, but, in the long term, could have decreased the abundances of species subject to florivory by decreasing recruitment (Anderson et al. 2001; Côté et al. 2004; Flaherty et al. 2018).

The increased abundance of white-tailed deer in the twentieth century has been associated with reductions of forb abundance in the understory of northern forests of North America (Côté et al. 2004; Wiegmann and Waller 2006). White-tailed deer have also been shown to consume flowers of *Polemonium vanbruntiae* in bogs (Flaherty et al. 2018), to consume flowers of *Iris hexagona* in a salt marsh (Geddes and Mopper 2006)*,* and to reduce the abundance of *Trillium* in forest understory (Augustine and Frelich 1998). A comparison of islands with and without deer found that the forb *Clintonia borealis* had fewer genets per ramet when deer were present (Palagi and Ashley 2019). In a prairie restoration when deer were hunted, the amount of browsed stems of *S. rigida*, which we also report as being deer browsed, decreased (Anderson et al. 2007). Deer florivory may not be exclusive to *Odocoileus virginianus* as Sitka deer (*Cervus nippon*) have also been reported to reduce flower cover causing subsequent decreases in pollinators (Sakata and Yamasaki 2015; Nakahama et al. 2020).

In contrast to forbs, we found greater abundance of the C3 grass species *P. oligosanthes* in unfenced plots and no effect of fencing on the abundance of the native C4 grass *S. nutans*, consistent with deer being browsers with minimal impact on grasses with intercalary meristems and low-nutrient tissues (Gordon and Herbert 2019; Bloodworth et al. 2020). We speculate that the greater biomass of *P. oligosanthes* outside the fence may be a response to deer trampling and/or increased light availability. *P. oligosanthes* was observed growing in the trampled vegetation where deer had slept. A similar long term study in a forest ecosystem in Wisconsin found that 18 years of deer exclosure caused increased abundance of forbs inside the fence, whereas grass abundance increased outside the fence (Rooney 2009). A follow-up study found that graminoids had low palatability to white-tailed deer (Begley-Miller et al. 2014). In our experiment, grasses were differentiated along an axis of N addition rather than fencing (Wedin and Tilman 1993). Rather than large increases in grass abundance when fences were removed, we found an increase in the forb *A. ludoviciana* outside the fence. *A. ludoviciana* has purported physical and chemical defenses that are tolerated by its specialist insect herbivore *Hypochlora alba* (Smith and Kreitner 1983), but presumably not by many other herbivores.

An interesting comparison can be drawn to a study at the Konza Prairie, where *Bison bison*, which preferentially graze on grasses such as *S. scoparium*, caused an increase in the cover of two forbs, Missouri goldenrod *Solidago Missourensis* and heath aster *Aster ericoides* (now *Symphyotrichum ericoides*) (Towne et al. 2005). In our study, deer reduced the abundance and flowers of two congeners of these species: *S. rigida* and *A. azureus* (now *S. oolentangiense*). Deer florivory might cause grassland communities to lose poorly defended forbs, whereas grazers such as *B. bison* consuming grasses might increase forb abundance. For example, our study reinforces a similar finding from the Konza Prairie that white-tailed deer have no detectable effect on dominant C4 grasses (Bloodworth et al. 2020). However, by explicitly looking at different forb species, we found that they could have markedly different responses to deer. We wonder if a more diverse community of mammalian herbivores, such as existed historically in the great plains of North America (Hartnett et al. 1997), might have maintained high plant diversity because of the differing effects of different types of herbivores on various species of both grasses and forbs (Ratajczak et al. 2022).

### Joint effects of N and deer florivory on plant coexistence

The responses of individual plant species to the experimental treatments suggest that some common forb species varied along a trade-off axis defined by N addition and fencing (Fig. 3). This effect was most clear for two dominant species. *S. rigida* was most abundant fenced in plots with no added N and was much rarer in unfenced plots that received high rates of N addition. *A. ludoviciana* had exactly the opposite responses to these treatment combinations, being rare if fenced and N was not added or added at a low rate. The markedly different responses of these two species were clearly visible in the field (Fig. 3c): *S. rigida* is the species with yellow flowers and *A. ludoviciana* is the species with silver-gray foliage and flowers. In this photo on the right, *S. rigida* is the dominant species within a 0.0 g N m^−2^ year^−1^ with all other nutrients inside the fence treatment. On the left, *A. ludoviciana* is the dominant species within a 9.52 g N m^−2^ year^−1^ with all other nutrients outside the fence treatment. Although we cannot state with certainty the underlying mechanism driving the changes in these species’ abundances, the reported herbivory defenses of *A. ludoviciana* (Smith and Kreitner 1983), and the reported strong N competitive ability of *S. rigida* (Table 1, Harpole and Tilman 2006) are consistent with them having a trade-off between N competitive ability and resistance to deer herbivory.

Alternatively, one could consider the possibility that light competition might structure this plant community (Borer et al. 2014; Eskelinen et al. 2022). There may exist a threshold along the gradient of N addition where soil fertility no longer limits productivity and water or light becomes a dominant limiting factor (Tilman 1985). Comparing the impacts of white-tailed deer browsing with bison grazing using the lighting experimental design of Eskelinen et al. (2022) could be informative in this ecosystem. At present, it is unclear if selective deer browsing may structure the plant community via increased light availability.

Our results suggest that florivory, and by extension other causes of seed predation, may be an important factor influencing the composition and diversity of grasslands, which has some conceptual similarity to the Janzen–Connell hypothesis (Janzen 1970). Theory suggests that an interspecific trade-offs between resource competition and herbivory can act as a coexistence mechanism in plant communities via a combination of apparent and exploitative competition (Holt et al. 1994). The Holt et al. (1994) theory examines this trade-off for two species competing for a single resource. Our multispecies system is more complex, but we do observe the interspecific competition versus herbivore susceptibility trade-off that Holt’s model assumed. In particular, *A. ludoviciana* increased in abundance outside the fence suggesting it is a better competitor in the presence of herbivory or has a lower R** (Holt et al. 1994). In contrast, *S. rigida* has been reported as having a low R* for nitrate (Table 1, Harpole and Tilman 2006) and also decreased in abundance when fences were removed (Fig. 3). While the Holt et al. (1994) model originally assumed herbivory to be broadly defined, in our case the herbivory might mainly be florivory. The consumption of seeds has also revealed a competition–defense trade-off in an annual plant community when granivorous ants preferentially consumed the seeds of species that had both larger seeds and were competitively dominant, albeit with mixed effects on coexistence (Petry et al. 2018). The results of Petry et al. (2018) and the present study suggest a testable hypothesis of the Holt et al. (1994) model that the consumption of flowers or seeds may be a form of herbivory that invokes a competition-defense trade-off (Viola et al. 2010).

### Study limitations

The data presented appear consistent with previous results at our site showing that deer are the main large mammalian herbivore (Ritchie and Tilman 1995; Ritchie et al. 1998; Knops et al. 2000). Our fencing likely did not exclude small mammals such as rodents and lagomorphs and we have no data on the role of small mammals in this experiment. When fencing has excluded small granivorous mammals in a desert plant community, grass abundance dramatically increased (Brown and Heske 1990; Brown 1998). While our data and observations and several examples in the literature suggest that deer florivory can structure forb abundances (Augustine and Frelich 1998; Geddes and Mopper 2006; Anderson et al. 2007; Sakata and Yamasaki 2015; Flaherty et al. 2018; Palagi and Ashley 2019; Nakahama et al. 2020), it is possible that other forms of herbivory explain the species' responses to the fencing treatment. It has also been suggested that some plant species that are susceptible to herbivory may display compensatory growth that could make them more abundant in the presence of herbivores (Augustine and McNaughton 1998). Because *A. ludoviciana* had the greatest positive response to the absence of fencing, it may merit further study in the tallgrass prairie ecosystem to see if its response is one of tolerance or resistance to deer and if it has chemical or physical defenses as Smith and Kreitner (1983) suggest. While the main detectable responses are for the most abundant species, rarer forbs and legumes trend in the same direction, but with statistically weak effects. It is plausible that florivory might induce propagule limitation for rarer forb species, but detection of such effects would require a great increase in sampling effort.

### Conclusions

We suggest that white-tailed deer, despite consuming a small portion of the total plant biomass, significantly impacted a tallgrass prairie ecosystem by preferentially consuming flowers of some dominant forb species such as *S. rigida* and thereby promoting a great increase in abundance of a competing forb, *A. ludoviciana*. Deer browsing, however, did not interact with N addition to significantly impact plant biomass. Furthermore, deer browsing did not offset the loss of plant biodiversity associated with added N, perhaps because the small amount of biomass removed by their browsing was insufficient to reverse N-caused light limitation (Borer et al. 2014; Eskelinen et al. 2022). In total, our results suggest that species-specific deer florivory may be a major factor impacting competition and the relative abundances of dominant forb species (Augustine and Frelich 1998; Anderson et al. 2001, 2007; Geddes and Mopper 2006; Flaherty et al. 2018; Palagi and Ashley 2019). While grasslands are named for their grasses and the study of their ecology has often focused on grazing herbivores, such as *Bison bison* (Knapp et al. 1999; Towne et al. 2005; Ratajczak et al. 2022), our experiment suggests that selective deer florivory can also impact plant species abundances and promote plant coexistence in grasslands via plant interspecific trade-off between resistance to deer florivory and competitive abilities for limiting resources.

### Supplementary Information

Below is the link to the electronic supplementary material.

Supplementary file1 (DOCX 41 kb)

## Data Availability

Raw data for re-use is available on the Environmental Data Initiative: Plant aboveground biomass (Tilman 2020); Counts of White-Tailed Deer Florivory (Furey and Tilman 2023). Derived summary data is available on Zenodo with the R code.

## Code availability

R Code is available on Zenodo via github at the following DOI: https://doi.org/10.5281/zenodo.8311045.
