# Supplementary material for: Trade-offs between deer herbivory and nitrogen competition alter grassland forb composition
Source: Oecologia. 2023 Dec 13;204(1):47–58. doi: 10.1007/s00442-023-05485-9 (PMC10830730; doi:10.1007/s00442-023-05485-9)
Supplement: Supplementary file 1 — Supplementary file1 (DOCX 41 kb) [file 442_2023_5485_MOESM1_ESM.docx]

**Supplemental Appendix**

*Title*: Tradeoffs between deer herbivory and nitrogen competition alter grassland forb composition

*Running title:* Tradeoffs between deer herbivory

*List of authors:* George N. Furey^1^ and David Tilman^1,2^

**Supplemental Figures**

Supplemental Fig. S1: Spatial layout of the experimental design. Each tile represents one plot labelled with its assigned plot number. (a) The layout of the fencing treatment: "Deer -" Fenced plots are shown in green. "Deer +" Unfenced plots are shown in orange. (b) The layout of the nitrogen addition treatment. There are 8 levels of added nitrogen (0.0, 1.02, 2.04, 3.4, 5.4, 9.5, 17.0, 27.2 g N m^-2^ yr^-1^) plus nutrients P, K, Ca, Mg, S and trace metals (Tilman 1987). There are two sets of control plots. Treatment I which received no nutrients of any kind and Treatment A which received no N, but all other nutrients. Treatment A serves as the control to test solely for the effect of N addition whereas Treatment I serves as the control for nutrient addition of any kind.

**Supplemental Tables**

Supplemental Table S1: Summary ANOVA table testing the dependance of the total live aboveground herbaceous biomass on the fencing treatment as a categorical variable, the nitrogen addition treatment as an unordered categorical variable and the natural log of year as a continuous variable for 11 years of data (2005-2019, not including 2012, 2013, 2016, 2017). n = 36.

| Term | numDF | denDF | F-value | *P*-value |
| --- | --- | --- | --- | --- |
| Fencing Treatment | 1 | 29 | 5.3 | 0.029 |
| Nitrogen Treatment | 5 | 29 | 6.9 | <0.01 |
| log(Year) | 1 | 359 | 142.2 | <0.001 |

Supplemental Table S2: Comparison of least square means of total live aboveground biomass (g m^-2^) across nitrogen addition treatments (g N m^-2^ yr^-1^). Means are averaged across fencing treatments. SE refers to standard error of the difference between each mean. P values were corrected using a tukey family-wise correction.

| Contrast  (g m^-2^ yr^-1^of N) | Difference | SE | DF | T.ratio | P value |
| --- | --- | --- | --- | --- | --- |
| 0.00 - 1.02 | -19.2 | 27.8 | 11 | -0.7 | 0.979 |
| 0.00 - 2.04 | -67.5 | 33.7 | 11 | -2.0 | 0.398 |
| 0.00 - 3.40 | -78.6 | 28.9 | 11 | -2.7 | 0.148 |
| 0.00 - 5.44 | -126.0 | 32.8 | 11 | -3.8 | **0.025** |
| 0.00 - 9.52 | -216.2 | 46.7 | 11 | -4.6 | **0.007** |
| 1.02 - 2.04 | -48.3 | 32.9 | 11 | -1.5 | 0.689 |
| 1.02 - 3.40 | -59.5 | 28.0 | 11 | -2.1 | 0.342 |
| 1.02 - 5.44 | -106.8 | 31.9 | 11 | -3.3 | 0.055 |
| 1.02 - 9.52 | -197.0 | 46.1 | 11 | -4.3 | **0.013** |
| 2.04 - 3.40 | -11.2 | 33.8 | 11 | -0.3 | 0.999 |
| 2.04 - 5.44 | -58.6 | 37.1 | 11 | -1.6 | 0.628 |
| 2.04 - 9.52 | -148.8 | 49.9 | 11 | -3.0 | 0.099 |
| 3.40 - 5.44 | -47.4 | 32.9 | 11 | -1.4 | 0.704 |
| 3.40 - 9.52 | -137.6 | 46.8 | 11 | -2.9 | 0.105 |
| 5.44 - 9.52 | -90.2 | 49.3 | 11 | -1.8 | 0.486 |

Supplemental Table S3: The effect size of separate linear mixed effects model testing the dependence of individual species biomass' on nitrogen as a linear variable and the fencing treatment as a categorical variable. Using years of data 2006-2019, not including years 2012, 2013, 2016, 2017. *P*-values were corrected using the False Discovery Rate correction (FDR). SE = standard error. The variable "Fencing" is coded such that a positive value means a gain in abundance outside the fence.

| Species | Effect of Nitrogen | Nitrogen SE | Nitrogen *P*-value | Nitrogen *P*-value (FDR) | Effect of Fencing | Fencing SE | Fencing *P*-value | Fencing *P*-value (FDR) |
| --- | --- | --- | --- | --- | --- | --- | --- | --- |
| Ambrosia coronopifolia | -0.39 | 0.55 | 0.485 | 0.5389 | 7.93 | 2.61 | 0.00460 | 0.0232 |
| Artemisia ludoviciana | 13.85 | 2.35 | 1.3E-06 | 1.3E-05 | 34.22 | 12.16 | 0.00820 | 0.0273 |
| Elymus repens | 10.66 | 3.57 | 5.3E-03 | 0.0175 | -13.09 | 21.56 | 0.54800 | 0.6089 |
| Euphorbia corollata | 0.11 | 0.20 | 0.585 | 0.585 | -6.93 | 1.89 | 0.00085 | 8.5E-03 |
| Lathyrus venosus | -0.51 | 0.52 | 0.3353 | 0.4789 | -6.40 | 3.29 | 0.06030 | 0.0861 |
| Panicum oligosanthes | -0.20 | 0.28 | 0.4847 | 0.5389 | 3.70 | 1.57 | 0.02410 | 0.0449 |
| Poa pratensis | 7.11 | 1.45 | 2.4E-05 | 1.2E-04 | -3.52 | 8.67 | 0.68780 | 0.6878 |
| Solidago rigida | -4.73 | 1.92 | 0.0194 | 0.0388 | -31.65 | 13.02 | 0.02070 | 0.0449 |
| Sorghastrum nutans | -0.44 | 0.18 | 0.0189 | 0.0388 | 1.34 | 1.09 | 0.22570 | 0.2821 |
| Symphyotrichum oolentangiense | -0.60 | 0.33 | 0.0759 | 0.1265 | -5.82 | 2.52 | 0.02700 | 0.0449 |
